# Rapid prototyping of all-solution-processed multi-lengthscale electrodes using polymer-induced thin film wrinkling

**DOI:** 10.1038/srep42543

**Published:** 2017-02-13

**Authors:** Christine M. Gabardo, Robert C. Adams-McGavin, Barnabas C. Fung, Eric J. Mahoney, Qiyin Fang, Leyla Soleymani

**Affiliations:** 1 McMaster University, School of Biomedical Engineering, Hamilton, L8S 4L7, Canada; 2 McMaster University, Department of Engineering Physics, Hamilton, L8S 4L7, Canada

## Abstract

Three-dimensional electrodes that are controllable over multiple lengthscales are very important for use in bioanalytical systems that integrate solid-phase devices with solution-phase samples. Here we present a fabrication method based on all-solution-processing and thin film wrinkling using smart polymers that is ideal for rapid prototyping of tunable three-dimensional electrodes and is extendable to large volume manufacturing. Although all-solution-processing is an attractive alternative to vapor-based techniques for low-cost manufacturing of electrodes, it often results in films suffering from low conductivity and poor substrate adhesion. These limitations are addressed here by using a smart polymer to create a conformal layer of overlapping wrinkles on the substrate to shorten the current path and embed the conductor onto the polymer layer. The structural evolution of these wrinkled electrodes, deposited by electroless deposition onto a nanoparticle seed layer, is studied at varying deposition times to understand its effects on structural parameters such as porosity, wrinkle wavelength and height. Furthermore, the effect of structural parameters on functional properties such as electro-active surface area and surface-enhanced Raman scattering is investigated. It is found that wrinkling of electroless-deposited thin films can be used to reduce sheet resistance, increase surface area, and enhance the surface-enhanced Raman scattering signal.

Highly conductive layers at the interface between solid devices and liquid samples are critical components of electrical bioanalytical sensors and transducers^1^,^2^,^3^,^4^,^5^. Borrowed from the microelectronics industry, vacuum-based vapor deposition techniques are commonly used to deposit conductive films onto the substrates of bioanalytical devices^6^,^7^. While precise control over the composition and thickness of deposited film can be achieved with these methods, they rely on expensive vacuum-based instrumentation placed in cleanrooms for depositing and patterning thin films. This model works for large volume production of microelectronics; however, there is a need for new manufacturing methods that are cost-effective at prototyping and low-volume production levels. These methods should also be able to be extend to large volume manufacturing for developing new classes of bioanalytical devices that rely on miniaturized and disposable components, such as chips, strips, and cartridges. Depositing and patterning electrodes using all-solution-processed methods, such as printing, enables device fabrication outside the semiconductor foundries and has been used for applications ranging from rapid prototyping to high volume production of bioanalytical devices, such as glucose monitoring strips^8^. However, the difficulty with printed bioanalytical electrodes for use in new and advanced applications, such as ultrasensitive biomolecular detection, is twofold. Firstly, these electrodes suffer from conductivity issues without high temperature post-printing steps which are incompatible with many polymer substrates needed for creating inexpensive disposable bioanalytical cartridges^9^. Secondly, it is difficult to create three-dimensional electrodes with controllable structural parameters that are critical for tuning the metrics of bioanalytical systems, such as sensitivity^10^,^11^, response time^12^,^13^, and power requirement^14^. To address the challenges involved in inexpensive fabrication of three-dimensional metallic electrodes for bioanalytical sensing, we sought to develop an all-solution processing method that (1) enabled the fabrication of high conductivity electrodes, capable of being patterned over large surface areas, without the need for high temperature processing, and (2) was suitable for the creation of three-dimensional metallic films with a high degree of structural tunability.

Electroless deposition is a facile and inexpensive solution-based method for producing high quality uniform and continuous metallic films on a variety of conductive and non-conductive substrates^15^. Although electroless deposition can create high quality conductive films, the resulting planar electrodes often lack functional micro-/nano-structures with significant height (>1 μm). Wrinkling through the compression of thin films in-plane presents a rapid and inexpensive method to controllably add multi-scale structuring to planar films while reducing their footprints, both features important for creating bioanalytical systems^16^,^17^,^18^,^19^,^20^,^21^. Thin film wrinkling using shrink memory films has been mainly applied to thin metallic films deposited using physical vapor deposition (PVD) methods^17^,^18^. More recently, researchers have explored other methods to deposit conductive/semi-conductive films for subsequent wrinkled structuring, such as depositing^22^, implanting, or spray-coating metallic nanoparticles^23^ or semiconductive nanocrystal films^24^ and spin-coating conductive polymer films^25^. Although these methods introduced novel nanostructuring and/or porosity not observed in the PVD films, they did not produce the uniformity, continuity, and conductivity of PVD films necessary for electrical device applications, like sensors, due to the low density and/or non-uniform deposition of the nanomaterials. Therefore, it is important to explore integration of thin film wrinkling with all-solution-processing methods, such as electroless deposition, that possess high degree of uniformity and continuity to create inexpensive and high quality, yet structurally tunable electrodes.

Here we demonstrate that patterned wrinkled electrodes with tunable minimum feature sizes, topography, and porosity can be readily fabricated using all-solution-processing on shrink memory polymer substrates. The resulting structurally tunable three-dimensional electrodes also exhibit a combination of useful functional properties including high surface area, good electrical conductivity, and significant enhancement in surface-enhanced Raman scattering signal, making them suitable for a range of chemical and bioanalytical sensing applications. In addition, since these wrinkled electrodes are fabricated using a combination of inexpensive solution-phase processes – molecular self-assembly, colloidal nanoparticle seed layer deposition, electroless deposition – they do not rely on expensive instrumentation and can be applied to rapid prototyping and be extended to batch-based industry-scale manufacturing.

## Results and Discussion

### Fabrication of Wrinkled All-Solution-Processed Gold

**Figure 1:**
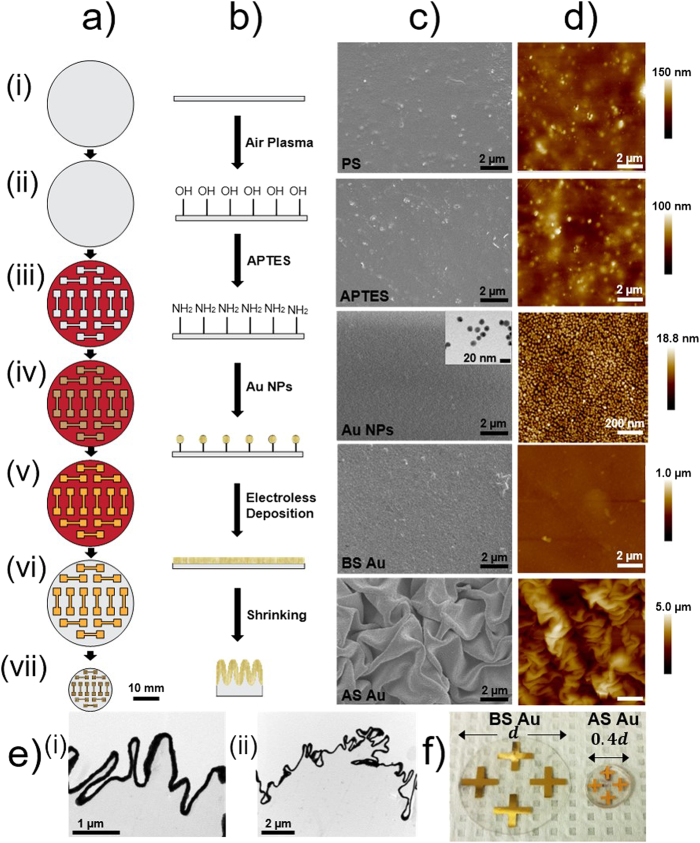
Fabrication of the all-solution-processed wrinkled films. (**a**) Schematic of the top view fabrication process: (i) Plasma cleaning pre-stressed PS substrate, (ii) modifying the substrate with an amino-silane (APTES) layer, (iii) masking the substrate with a xurography-patterned vinyl film, (iv) creating Au NP seed layer, (v) electroless deposition of Au, (vi) mask removal, and (vii) heating the device to create wrinkled electrode structures. (**b**) Side view schematic demonstrating the surface composition during the fabrication process. (**c**) SEM images after each fabrication step, inset of iv) displays TEM image of unbound as-synthesized Au NPs. (**d**) AFM images after each fabrication step. (**e**) TEM images of wrinkled film cross-sections showing the primary and secondary wrinkling at i) high and ii) low magnification. (**f**) Photograph of all-solution-processed electrodes before shrinking (BS) and after shrinking (AS), *d* indicates the diameter of the substrate.

Figure 1a,b schematically illustrates the all-solution-based fabrication process used to produce wrinkled gold structures to be used as electrodes or other sensing devices. Gold (Au) was chosen for this study due to its importance in chemical and biological sensing owed to its high conductivity, nobility/chemical inertness, and ability to be functionalized with thiol-terminated biorecognition molecules^26^,^27^. In this method, a biaxially pre-stressed commercially-available polystyrene (PS) substrate is treated with air plasma (Fig. 1ai) for surface activation and subsequent silanization (the surface chemistry of devices is shown in Fig. 1b). The activated PS substrate is incubated in a 10% (3-aminopropyl) triethoxysilane (APTES) solution (Fig. 1aii), which forms a molecular linking layer for connecting citrate-stabilized gold nanoparticles (~12 nm diameter) to the polystyrene substrate through electrostatic interactions (Fig. 1b). An adhesive vinyl shadow mask, patterned through xurography, is then bound to the substrate (Fig. 1aiii). The masked and functionalized PS substrates are then incubated in an Au nanoparticle (Au NP) solution to form a thin, uniform seed layer of Au NPs (Fig. 1aiv,b). In order to create high conductivity electrodes, electroless deposition is subsequently performed on the seed layer to produce a continuous gold film. (Fig. 1av)^28^. The PS substrate modified with a Au NP seed layer is immersed in a cyanide-free and environmentally-friendly plating solution containing the gold precursor (chloroauric acid (HAuCl_4_)) and the reducing agent (hydrogen peroxide (H_2_O_2_)). The monolayer of Au NPs on the PS acts as the nucleation site for the growth and formation of the continuous Au layer (Fig. 1b), and is expected to minimize the reduction of gold within the bulk solution. Following electroless deposition, the vinyl shadow mask is removed (Fig. 1avi), exposing the electrode geometry. The pre-stressed polymer substrate then undergoes a thermally induced shrinking process by heating it in an oven at 160˚C, past the glass transition temperature for PS, for 3 minutes. Heating the pre-stressed PS over the glass transition temperature reduces the length and width of the substrate by more than 60% and increases its thickness by over 600%^17^. As the polymer substrate contracts, it exerts a compressive stress on the overlaying Au film, driving the gold film to buckle and wrinkle and causing the lateral dimensions of the gold pattern to be reduced by 60% (Fig. 1avii).

Figure 1c,d demonstrates the structural evolution of all-solution-processed gold films after each processing step using scanning electron microscopy (SEM) (Fig. 1c) and atomic force microscopy (AFM) (Fig. 1d). The Au NPs bind and form a uniform layer on the silanized PS substrate, as evidenced in the corresponding SEM image and AFM measurements. The subsequent electroless deposition forms a continuous layer of Au over the substrate. We hypothesized this all-solution-processed gold layer to wrinkle similar to the previously reported sputtered films^17^,^18^ upon heating because of the mismatch between the elastic moduli of the stiff thin film and the compliant PS substrate. Following the heating of the pre-stressed PS substrate, we observed wrinkles that result in feature sizes in the nano/microscale and height variations in the micrometer scale. The high and low magnification TEM images of a cross-section of a wrinkled electrode (Fig. 1e) show the conformal interface between the Au layer and PS, as well as the primary (smaller) and secondary (larger) wrinkling along this interface. A photograph of devices before (BS) and after (AS) shrinking are displayed in Fig. 1f. It is apparent that the after shrinking, Au electrodes show the same electrode layout as the before shrinking Au electrodes; however, they appear miniaturized and show a darkened gold colour that is less reflective due to the wrinkled structuring of the electrodes (Fig. 1f) and Fig. S1. Moreover, the shrinking occurs across the entire substrate, reducing the lateral dimensions of the device to 40% of its initial sizes.

The shrinking of the polymer substrate and the resulting wrinkled Au layer has several physical advantages over the planar before shrinking films, including a reduction in the lateral dimensions of the patterned Au film, structuring of the Au layer, and improved film-to-substrate adhesion. The lateral reduction in size allows for development of smaller electrode geometries and smaller device footprints. With this xurography-based patterning method, 100 μm line-and-space arrays could be produced, as well as 200 μm diameter circular features^17^. When a scotch tape adhesion test was performed on films before and after this shrinking process, it was observed that the Au layer partially peeled off prior to shrinking, but could not be removed after the shrinking and wrinkling process. This improved adhesion can be attributed to the effective anchoring of the Au film within the primary and secondary PS wrinkles, as observed in the cross-sectional TEM images.

### Characterization and Tunability of Gold Films

**Figure 2:**
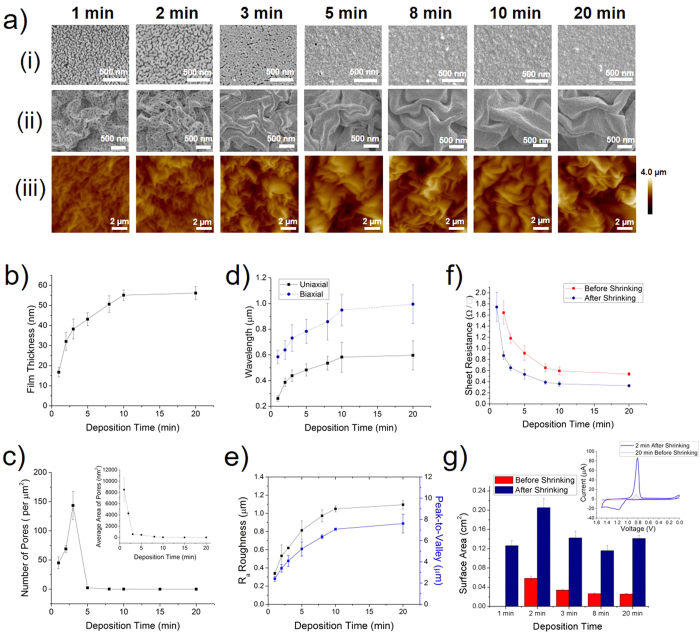
Structural evolution and characterization of Au films at different electroless deposition times. (**a**) SEM images of films (i) before shrinking and (ii) after shrinking, as well as (iii) AFM images of wrinkled electrodes. (**b**) AFM measured film thickness before shrinking. (**c**) Estimation of pore frequency in the all-solution-processed films before shrinking using SEM images, with the inset showing the average area of the pores. (**d**) Wrinkle wavelengths of uniaxially and biaxially wrinkled devices, as estimated by 2D FFT image analysis of SEM images. (**e**) Electrode roughness (R_a_) and peak-to-valley (PV) measured using white light interferometry. (**f**) Sheet resistance of electrode devices before and after shrinking. (**g**) Electrochemically determined surface area of electrode devices before and after shrinking. Inset displays the typical cyclic voltammograms of 2 min after shrinking device and 20 min before shrinking planar device in 0.05 H_2_SO_4_ with respect to Ag/AgCl at a scan rate of 0.05 V/s.

The ability to structurally tune the newly developed all-solution-processed wrinkled film is highly desirable for optimizing the functional parameters of these materials^10^. To achieve tunability in minimum feature size, porosity, thickness, and topography, we investigated the structure of the all-solution-processed wrinkled films fabricated using different electroless deposition durations (Fig. 2a–e). SEM images of the Au films before shrinking (Fig. 2ai) indicate that the film morphology strongly depends on the electroless deposition duration. The film continuity and porosity was strongly affected by deposition duration. Discontinuous islands of gold can be observed after 1 min of electroless deposition, while the coalescence between these islands increases as the electroless deposition duration increases until around 8 min, where a continuous gold film can be observed. This type of film growth, from separate islands to a complete layer through coalescence and percolation has been reported before in thin film systems^29^. Moreover, it was found that the film thickness increased with increasing deposition duration (Fig. 2b), with a plateau at approximately 55 nm observed around 10 min. While the concentrations of the precursors are decreasing with time, the largest contributor to the decrease in the gold deposition rate over time is likely the decrease in the surface free energy of the film as it becomes completely continuous^30^. Image analysis of these films (Fig. 2c) indicate that the density of pores increases between deposition durations of 1 and 3 min, as the large pores between islands are split into multiple smaller pores (average size of pores shown in Fig. 2c inset), then decreases rapidly until 8 min.

It is evident that after shrinking, the wrinkle size and porosity are also dependant on the electroless deposition duration (Fig. 2aii). Interestingly, an additional degree of tunability in material structure is observed through this newly-developed all-solution-processing method that was not previously reported with wrinkled PVD films. Thin films deposited at short electroless deposition durations, demonstrating a non-continuous and porous structure, are able to wrinkle and become more connected during the shrinking process.

Based on the SEM images of the wrinkled films (Fig. 2aii), the wrinkle sizes appear to increase with increasing electroless deposition duration. To quantify the all-solution-processed wrinkle wavelengths, we took the two-dimensional fast Fourier transform (2D FFT) (Fig. 2d) of the SEM images of films wrinkled biaxially, as well as uniaxially, created by physically restricting the shrinking process to one axis (Fig. S2). The analysis of uniaxial and biaxial wrinkle wavelengths demonstrates that the wavelength distribution broadens and shifts towards larger values with increasing deposition duration until 10 min, with larger wavelength values observed for biaxial wrinkles compared to uniaxial wrinkles of films of similar deposition times/thickness. The dependence of wrinkle wavelength on film thickness is in agreement with theoretical models and experiments performed with PVD thin films^31^,^32^,^33^. When the average wrinkle wavelength is plotted against the average film thickness (Fig. S3), an increasing, but not perfectly linear trend is observed. Previous theoretical models indicate that the uniaxial wrinkle wavelength is directly proportional to the thin film thickness *and* the cubic root of the ratio of the elastic moduli of the thin film and the substrate, suggesting that the mechanical properties of the film is also changing with varying electroless deposition times. The deviation from the linear trend is observed to more pronounced for biaxial wrinkled films, which can be attributed to the wavelength being more strongly dependent on the mechanical properties of the film, scaling with the cubic root *squared* of the ratio of the elastic moduli of the thin film and the substrate^18^.

The AFM data demonstrates that the thin film topography is also tunable by varying the duration of electroless deposition before wrinkling. As the electroless deposition duration is increased, we see a corresponding increase in topographical variation across the wrinkled films (Fig. 2aiii). The roughness of the surface of the wrinkled Au layer is also characterized using white light interferometry (Fig. 2e). With increasing deposition time, the surface roughness (R_a_) and peak-to-valley (PV) variation increase rapidly until 10 min. Between 10 to 20 min, there is a smaller increase in the surface roughness and PV values, consistent with the finding that the film thickness plateaus after 10 min. of electroless deposition.

In order to study how the structural differences between electrodes translate into differences in their electrical properties, we performed four-point probe sheet resistance measurements before and after shrinking (Fig. 2e). It is evident that the sheet resistance of the electrodes, before and after shrinking, decreases rapidly as the deposition duration increases between 1 and 10 min, and then a slower decrease in sheet resistance is observed as the deposition duration increases past 10 min. The rapid decrease observed prior to 10 min is attributed to the filling of the porous structures and the increasing film thickness during this stage of deposition. Moreover, it is observed that the electrical properties of the electrodes are improved through the shrinking process at all deposition durations, as the sheet resistance is significantly lower after shrinking. The decrease in sheet resistance after shrinking can be attributed to the increased connectivity between adjacent folds and the increased overall conductor path thickness, as well as partially or fully connecting discontinuities in the porous film present at short deposition times. It should be noted that devices that were fabricated using 1 min of electroless deposition were not conductive enough to obtain sheet resistance values prior to shrinking, however the wrinkled electrodes at this deposition time did overcome the measuring threshold to obtain sheet resistance values. In addition, a comparison of all-solution-processed devices (8 min wrinkled, 0.39 ± 0.04 Ω/□) to sputter coated devices (~50 nm wrinkled, 0.40 ± 0.02 Ω/□) of approximately the same thickness was performed and the electroless deposited wrinkled devices of the same dimensions had approximately the same resistance of the sputter coated devices (Fig. S4). All-solution-processing has been previously used to fabricate conductive biocompatible polymer surfaces with optical transparency for smart cell scaffolding^25^ applications. However, the all-solution-processed films presented here demonstrate sheet resistances as low as 0.25 Ω/□, which is significantly lower (by > 1000 times) compared to the previously-developed conductive polymer-based surfaces. This makes the demonstrated electrodes ideally suited for electrical/electrochemical sensing applications requiring high conductivity.

High surface area electrodes are finding applications in a number of fields, ranging from biosensing^34^,^35^ to energy storage and conversion^36^. To assess the suitability of the all-solution-processed Au films for use as high surface area electrodes in electrochemical applications, we measured their electrochemical response using cyclic voltammetry (Fig. S5) and examined their electro-active surface areas (Fig. 2g). Electrodes deposited for 1 min, 2 min, 3 min, 8 min, and 20 min having the same geometric surface area (0.028 cm^2^) were evaluated before and after shrinking using cyclic voltammetry in dilute sulfuric acid (Fig. S5). It is evident from the voltammograms that shrinking increases the magnitude of the peak attributed to the reduction of gold oxide (at approximately 0.81 V) for all electrodeposition durations. It is further observed that the structural characteristics of the electrodes, such as porosity and roughness – tuned by the deposition duration – result in varying reduction peak magnitudes. Furthermore, the porous and wrinkled electrodes deposited for 2 min demonstrated the largest reduction peak magnitude within the examined dataset (Fig. S5 and Fig. 2g-inset). The electroactive surface areas of the electrodes were obtained by integrating the area under the reduction peak (details in methods section) and summarized in Fig. 2g. The surface area of the electrodes deposited for 8 min and 20 min before shrinking were approximately equal to their projected surface area. However, the measured surface area of the 2 min and 3 min devices before shrinking were larger by 106% and 20%, respectively, compared to their geometric surface areas. This is attributed to the presence of the pores within the electrode film (Fig. 2a) at shorter deposition durations. The electroactive surface area of the 1 min before shrinking device could not be determined due to insufficient conductivity. All the wrinkled electrodes showed significantly larger electroactive surface areas compared to their before shrinking counterparts for the same constrained area (0.028 cm^2^). This is expected and consistent with previous results obtained using PVD films because the shrinking process wrinkles the electrode material into a fraction (~16%)^17^ of the original footprint. The 2 min electroless deposited device demonstrated the largest surface area after shrinking (0.204 cm^2^) corresponding to a greater than 7 times enhancement compared to the geometric surface area. The decrease in surface area at 3 min (0.142 cm^2^) and even further at 8 min (0.116 cm^2^) compared to the 2 min devices is due to the partial and complete filling of the porous features within the Au films as observed in the SEM images (Fig. 2a). The surface area of the 20 min after shrinking devices was larger than the 8 min after shrinking devices, which is hypothesized to be related to the more separated and accessible wrinkles present in the 20 min devices. It is clear that the electroactive surface areas of the all-solution-processed electrodes can be precisely controlled by tuning the thin film porosity and wrinkle sizes, which is enabled by varying the duration of electroless deposition before electrode wrinkling.

### Application of Gold Films to Surface Enhanced Raman Spectroscopy

**Figure 3:**
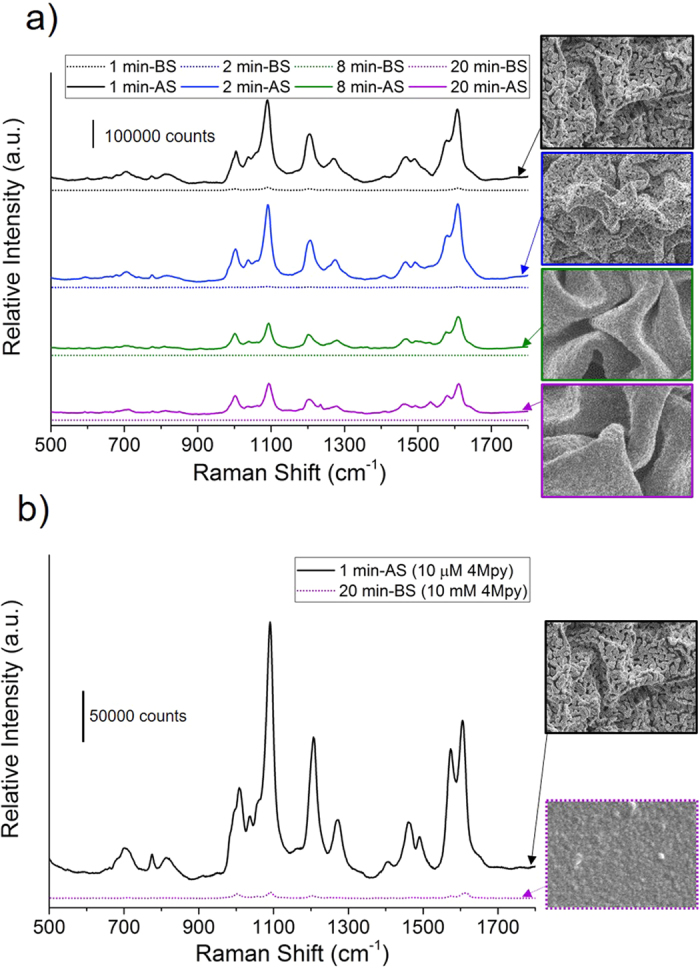
Application of Au films as surface enhanced Raman spectroscopy substrates. (**a**) SERS spectra of Au films at different electroless deposition durations before shrinking (BS) and after shrinking (AS) after 1 hour incubation with 1 mM 4 Mpy. (**b**) SERS spectra after 1 hour incubation of 20 min-BS with 10 mM 4 Mpy and 1 min-AS with 10 μM 4 Mpy used to calculate the enhancement factor.

In addition to their role as electrodes, nanostructured metal films play an important role as substrates for enabling surface enhanced Raman spectroscopy (SERS)^37^,^38^. Due to the presence of nano-gaps, sharp edges, and sharp corners on nanostructured noble metal substrates, very large electromagnetic (EM) fields can be produced by exciting the surface plasmon resonance at the surfaces of substrates with light^39^,^40^,^41^. As a proof-of-concept SERS sensor, Au films at different electroless deposition durations (1 min, 2 min, 8 min, and 20 min) were applied as substrates for the sensing of 4-mercaptopyridine (4 Mpy), and the spectra were measured before and after shrinking (Fig. 3a). The 4 Mpy is incubated on the before shrinking substrates, and it is evident from the SERS spectra that the intensity of spectral features becomes larger after shrinking. This can be attributed to the sharp Au film features that are produced through the wrinkling process, as well as the increase in the surface density of the analyte as the device footprint is reduced. Although the wrinkling enhances the Raman signal for all four deposition durations, the enhancement is more significant for the continuous films (8 min and 20 min), where statistically significant peaks are only evident after wrinkling, and likely due to the creation of EM hot spots. On the other hand, the porous films have nanostructures that can contribute to the Raman signal enhancement even prior to wrinkling, as evidenced by the detectible peaks in their before shrinking spectra. The wrinkled 1 min and 2 min devices show higher intensity spectra (2.5 to 3.3 times larger) compared to the wrinkled continuous films (8 min and 20 min) after shrinking, likely due to the high density of pores and nano-gaps within the wrinkled film that could be attributed to the EM field enhancement and their higher surface areas. Due to the very low peaks (not statistically different than the background noise) of the continuous planar films, the enhancement factor of the porous wrinkled films compared to planar gold films was determined by comparing 1 min substrates after shrinking incubated with 10 μM 4 Mpy against 20 min substrates before shrinking incubated with 10 mM 4 Mpy (Fig. 3b). The 1090 cm^−1^ peak associated with ring stretching/breathing and C-S stretching was used to calculate the enhancement factor (equation in Experimental Section). The 1 min and wrinkled substrates were able to enhance the signal over planar Au devices (20 min-BS) by 4.24 × 10^4^. Accounting for the larger surface area of the 1 min wrinkled device over the planar device, the enhancement in the Raman signal, discounting the role of analyte concentration, is 8.54 × 10^3^. Evidently, the structural tunability of these all-solution-processed metallic films allows them to be extended to applications where nanostructuring and high surface area are crucial to sensor performance.

## Conclusion

In summary, we have developed a rapid all-solution-processing method by combining electroless deposition and thin film wrinkling on shrink-memory polymer substrates. Through this work, we have: (1) demonstrated the tunability of the resulting Au film in terms of porosity, topography and morphology through the electroless deposition parameter manipulation and shrink-induced wrinkling; (2) characterized the effect of the structural control of the metallic film on important functional parameters, such as the conductance and electrochemically active surface area; and (3) translated the structural control to functional optimization of substrates for surface enhanced Raman spectroscopy. As a result, we have discovered that electroless deposition duration and wrinkling are key parameters for creating functional three-dimensional wrinkled materials that are optimized in terms of surface area, morphology, conductance, and SERS enhancement for use in application-specific devices. With the speed, ease, and low cost per surface area that these highly conductive functional films are fabricated, we foresee them being applicable to many electrochemical (biosensors, energy storage and conversion devices) and optical (surface plasmon resonance, surface enhanced Raman biosensing) applications.

## Methods

### Chemicals

(3-Aminopropyl) triethoxysilane (APTES, 99%), 4-Mercaptopyridine (4 Mpy, 95%), hydrogen tetrachloroaurate(III) trihydrate (HAuCl_4_ · 3H_2_O, > 99.9%), and hydrogen peroxide (H_2_O_2_, 30%) were purchased from Sigma-Aldrich (St. Louis, Missouri). Sulfuric acid (H_2_SO_4_) was purchased from Calden (Georgetown, Ontario). All reagents were of analytical grade and were used without further purification. Milli-Q grade water (18.2 M Ω) was used to prepare all solutions.

### Polystyrene Substrate Preparation

Pre-stressed polystyrene (PS) substrates (Graphix Shrink Film, Graphix, Maple Heights, Ohio) were cut into the desired shape using the Robo Pro CE5000- 40-CRP cutter (Graphtec America Inc., Irvine, CA). The PS substrates were cleaned with 2-propanol, DI H_2_O, and then dried with air. The substrates were treated with air plasma in an Expanded Plasma Cleaner (Harrick Plasma) on HIGH RF power setting for 60 seconds, oxidizing the surface of the substrate. Then, the substrates were immersed in a 10% APTES solution in an Incubating Mini Shaker (VWR International) for 16 hours at 120 rpm and at room temperature. Following silanization, the substrates were sonicated in DI H_2_O for 10 minutes, rinsed and dried. The desired electrode layout was designed in Adobe Illustrator and cut into vinyl (FDC 4304, FDC graphic films, South Bend, Indiana) using the Robo Pro CE5000- 40-CRP vinyl cutter. The masks were applied to the silanized PS substrates.

### Gold nanoparticle synthesis and deposition

Gold nanoparticles (Au NPs) were synthesized according to Preparation 1^42^ and were refrigerated until used. The masked PS substrates were then fixed in petri dishes using double sided tape and covered in a solution of Au NPs for 16 hours.

### Electroless Deposition

The Au NP covered PS substrates were placed in a 5 ml solution of 0.1% HAuCl_4_ solution on a shaker at 250 rpm at room temperature. Then, 250 μL of 30% H_2_O_2_ was added to the solution, initiating the electroless Au deposition. Bubbles forming on the edges of the vinyl mask were eliminated using a pipette tip. The vinyl mask was removed after deposition and the devices were then placed in an oven (Model: 664, Fisher Scientific, Marietta, OH) at 160˚C to shrink for 3 minutes.

### Electron Microscopy

SEM images of the gold films before and after shrinking were obtained using a JEOL JSM-7000S scanning electron microscope with an accelerating voltage of 2 kV, working distance of 6 mm, and low probe current. Pore size and wavelength data were extracted from SEM images using Image J and radially averaged 2D FFT analysis in MATLAB, respectively. TEM imaging was performed on cross-sections of the wrinkled devices using the JEOL 2010 F. These devices were embedded and sectioned by microtome prior to imaging.

### Sheet Resistance Measurements

Sheet resistance values of both shrunk and unshrunk electroless deposited electrodes on PS were measured with the van der Pauw method using the HL5500PC- Hall effect measurement system with a HL5500 buffer amplifier (Nanometrics, Milpitas, California). A Greek cross electrode geometry was used with a length and height of 10 mm before shrinking, and width of 3 mm.

### Adhesion Tests

Adhesion was assessed using the scotch tape peel test with the Instron 4411 (Instron Corporation, Norwood, MA, USA) machine. Tape was applied to the surface of the electrodes and peeled away using a constant velocity of 20 mm/min over multiple devices (n = 3).

### Roughness and Peak-to-Valley Measurements

The roughness (Ra) and peak-to-valley (PV) values of the wrinkled electrode devices were measured using a Zygo NewView 5000 white light interferometer (Zygo Corporation, Middlefield, CT) over multiple areas per device (n > 3) and over multiple devices (n = 3). Measurements were obtained with 10 × objective and 2 × image zoom setting (360 μm × 270 μm field of view) using a charge couple camera with an imaging pixel size of 11.2 μm. Data filtering and analysis was performed with MetroPro. A FFT high pass filter was applied, using a cut-off frequency 183.35 mm^−1^, and spikes with height values above 10 × RMS (root mean square) were removed to eliminate any variations due to the unevenness of the substrate or any surface contaminants, respectively.

### Electrochemistry

Electrochemistry was performed using a CHI 660D electrochemical workstation (CH Instrument, Austin, Texas) and a standard three-electrode set-up. The electrochemical system consisted of an Ag/AgCl reference electrode, a platinum wire counter electrode, and the all-solution-processed Au electrode as the working electrode. Electrochemical surface area measurements were performed in a 0.05 M H_2_SO_4_ solution at 0.05 V/s. The peaks for the reduction portion of the resulting cyclic voltammograms were integrated to obtain the electric charge involved in the redox process and divided by the surface charge density of Au (386 μC/cm^2^)^43^ to acquire the values of surface area.

### Raman Measurements

Raman measurements were performed using Renishaw inVia Raman Microscope, with a 632.8 nm HeNe laser at 5% power (25 mW) and 50x objective. Measurements were acquired using 10 s exposures and accumulated 5 times. Au film samples were incubated with 4 Mpy for an hour at room temperature, rinsed with DI H_2_O, and dried. Measurements were performed on samples prior to incubation with 4 Mpy, after incubation with 4 Mpy, and after shrinking. The background fluorescence signal was subtracted from the spectra and a fourth order polynomial baseline subtraction was performed. The enhancement factor (EF) of the 1 min-AS films was calculated with respect to the 20 min-BS films using Equation (1):


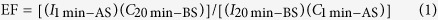


where *I* is the intensity of the 1090 cm^−1^ peak, which is associated with ring stretching/breathing and C-S stretching, and *C* is the concentration of 4 Mpy the substrate is incubated with.

## Additional Information

**How to cite this article:** Gabardo, C. M. *et al*. Rapid prototyping of all-solution-processed multi-lengthscale electrodes using polymer-induced thin film wrinkling. *Sci. Rep.* **7**, 42543; doi: 10.1038/srep42543 (2017).

**Publisher's note:** Springer Nature remains neutral with regard to jurisdictional claims in published maps and institutional affiliations.

## Supplementary Material

Supplementary Information
