# Supplementary material for: Rapid prototyping of all-solution-processed multi-lengthscale electrodes using polymer-induced thin film wrinkling
Source: Sci Rep. 2017 Feb 13;7:42543. doi: 10.1038/srep42543 (PMC5304207; doi:10.1038/srep42543)
Supplement: Supplementary Information [file srep42543-s1.pdf]

Supplementary Information

Rapid prototyping of all-solution-processed multi-lengthscale electrodes using polymer-induced thin film wrinkling

Christine M. Gabardo[1], Robert C. Adams-McGavin[2], Barnabas C. Fung[2], Eric J. Mahoney[1], Qiyin Fang[1,2], and Leyla Soleymani[1,2,*]

[1]McMaster University, School of Biomedical Engineering, Hamilton, L8S 4L7, Canada
[2]McMaster University, Department of Engineering Physics, Hamilton, L8S 4L7, Canada
[*]E-mail: soleyml@mcmaster.ca

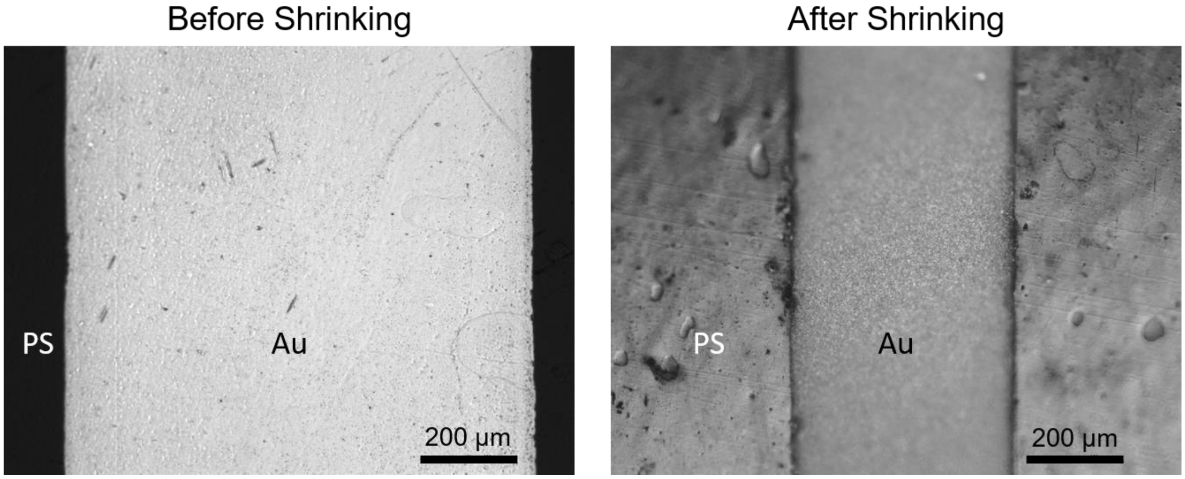

Figure S1. Patterned Au film line before and after shrinking to demonstrate the miniaturization of the pattern, as well as the texturing of the Au layer after shrinking.

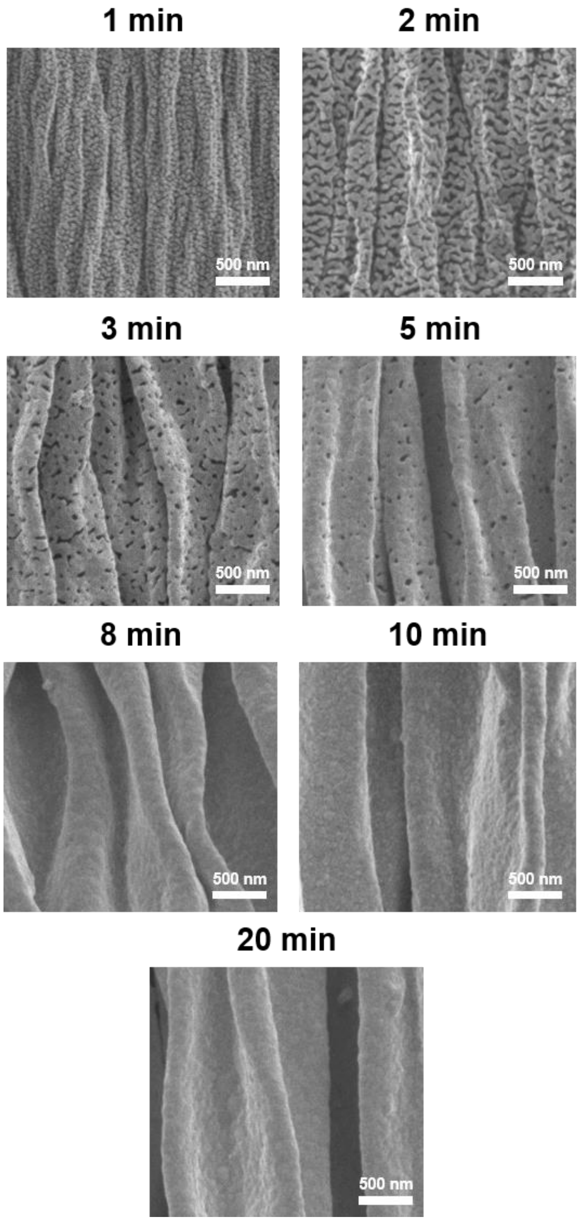

Figure S2. SEM images of uniaxial wrinkled Au films at different electroless deposition times.

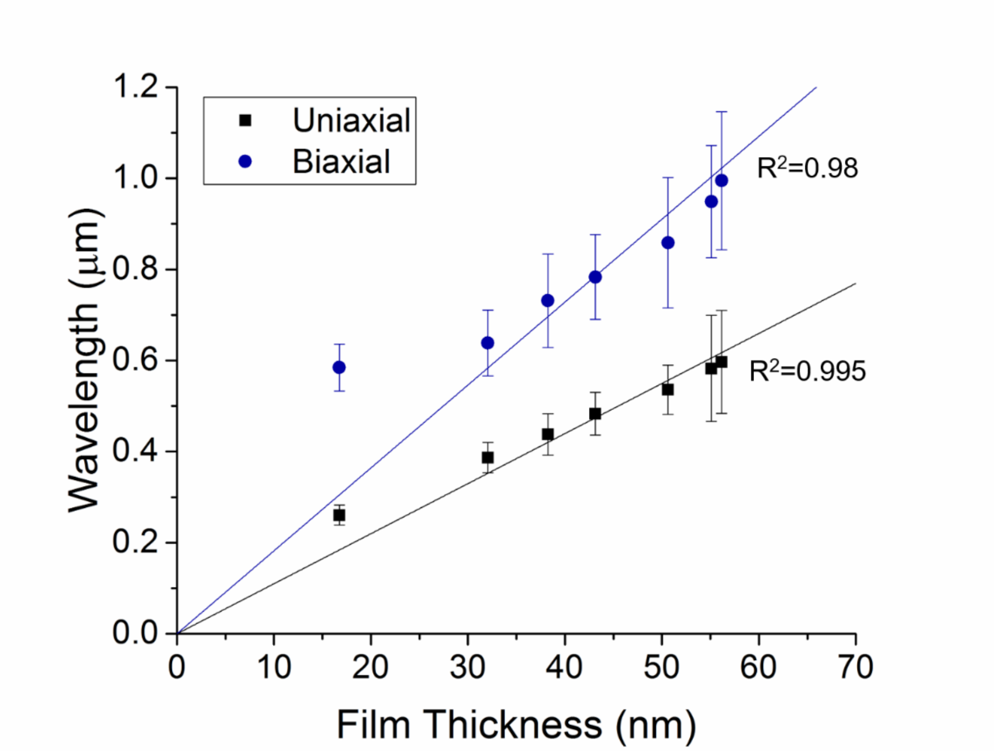

Figure S3. Electroless Au film thickness before shrinking and the resulting wavelength after shrinking.

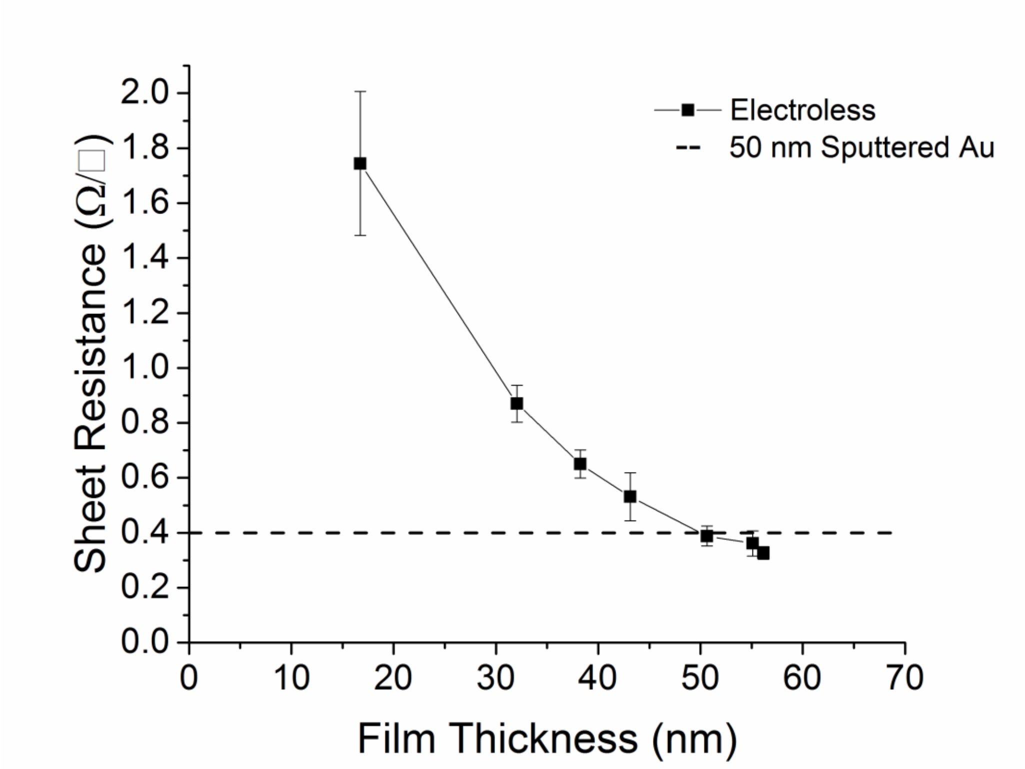

Figure S4. Sheet resistance measurements of wrinkled electroless deposited films with respect to film thickness compared to 50 nm wrinkled sputtered Au film.

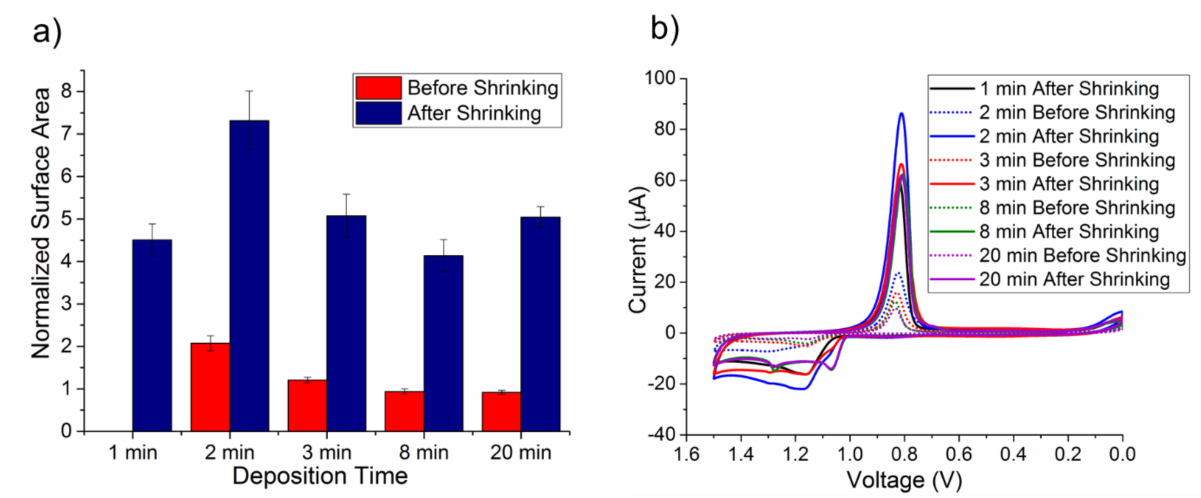

Figure S5. Electrochemical characterization of electrode devices. a) Normalized surface area (electroactive surface area/ geometric surface area) for devices deposited at varying durations before and after shrinking. b) Representative cyclic voltammograms (with respect to Ag/AgCl reference electrode, scan rate 0.05 V/s) in dilute H_2SO_4 (0.05 M) for devices deposited at varying durations before and after shrinking. The data for 1 min before shrinking device is not provided due to insufficient conductivty for performing cyclic voltammetry.
